# 

**Published:** 1900-09

**Authors:** 


### Society Notes.

### The Mississippi Valley Medical Association.

We have received the preliminary program of the meeting of this society to be held at Asheville, N. C., October 9, 10 and 11, prox. Dr. I. N. Love will deliver the address in medicine. Drop a card to Dr. H. E. Tully, St. Louis, secretary, for information if you wish to attend the meeting. Dr. H. N. Moyer, Chicago, is president. We received program too late to publish in full.

